# Machine learning to assist clinical decision-making during the COVID-19 pandemic

**DOI:** 10.1186/s42234-020-00050-8

**Published:** 2020-07-10

**Authors:** Shubham Debnath, Douglas P. Barnaby, Kevin Coppa, Alexander Makhnevich, Eun Ji Kim, Saurav Chatterjee, Viktor Tóth, Todd J. Levy, Marc d. Paradis, Stuart L. Cohen, Jamie S. Hirsch, Theodoros P. Zanos, the Northwell COVID-19 Research Consortium, Lance B. Becker, Jennifer Cookingham, Karina W. Davidson, Andrew J. Dominello, Louise Falzon, Thomas McGinn, Jazmin N. Mogavero, Gabrielle A. Osorio

**Affiliations:** 1 Institute of Bioelectronic Medicine, Feinstein Institutes for Medical Research, Northwell Health, Manhasset, NY USA; 2 Institute of Health Innovations and Outcomes Research, Feinstein Institutes for Medical Research, Northwell Health, Manhasset, NY USA; 3 Donald and Barbara Zucker School of Medicine at Hofstra/Northwell, Northwell Health, Hempstead, NY USA; 4 Department of Information Services, Northwell Health, NYC Metro Area, NY USA; 5 Cardiology, Long Island Jewish Medical Center and Feinstein Institutes for Medical Research, Northwell Health, Manhasset, NY USA; 6 Holdings and Ventures, Northwell Health, Manhasset, NY USA

**Keywords:** Artificial intelligence (AI), Clinical decision-making, Coronavirus disease 19 (COVID-19), Healthcare, Machine learning (ML)

## Abstract

**Background:**

The number of cases from the coronavirus disease 2019 (COVID-19) global pandemic has overwhelmed existing medical facilities and forced clinicians, patients, and families to make pivotal decisions with limited time and information.

**Main body:**

While machine learning (ML) methods have been previously used to augment clinical decisions, there is now a demand for “Emergency ML.” Throughout the patient care pathway, there are opportunities for ML-supported decisions based on collected vitals, laboratory results, medication orders, and comorbidities. With rapidly growing datasets, there also remain important considerations when developing and validating ML models.

**Conclusion:**

This perspective highlights the utility of evidence-based prediction tools in a number of clinical settings, and how similar models can be deployed during the COVID-19 pandemic to guide hospital frontlines and healthcare administrators to make informed decisions about patient care and managing hospital volume.

## Background

Coronavirus disease 2019 (COVID-19), caused by the severe acute respiratory syndrome coronavirus 2 (SARS-CoV2), has spread to the level of a global pandemic (World Health Organization 2020) with, at the time of writing, over 1.6 million reported cases in the United States and 5.6 million worldwide. In the United States, New York City was the epicenter of the disease with over 199,000 confirmed cases and over 16,400 deaths (Center for Systems Science and Engineering (CSSE) at Johns Hopkins University 2020). At the pandemic’s peak, existing medical facilities were overwhelmed, with emergency departments (EDs), floor units, and Intensive Care Units (ICUs) stretched beyond capacity and resources (Evans and Armour 2020). Due to the challenges of this novel disease, healthcare providers, patients, and their families have been required to rapidly make crucial and difficult decisions with limited information. The phenotypes of COVID-19 range from no or relatively mild symptoms and uneventful recovery to rapid deterioration, acute respiratory distress syndrome (ARDS), multi-organ system failure, and death. The trajectory for patients most likely to decompensate is being investigated but remains elusive at present; lack of standardized care is forcing unprecedented workflow for physicians and nurses. Given the gravity of these circumstances and increase in the number of cases, there is a pressing need for tools that can augment current healthcare resources. Machine learning (ML) and artificial intelligence (AI) methods can be applied to understand subgroups of patients, guide clinical decision-making, and improve both operation- and patient-centered outcomes. This perspective highlights the benefits of these tools observed at various clinical settings and describes how the value of ML and AI algorithms, when conscientiously built, may be augmented during the COVID-19 pandemic.

## Main text

Identifying underlying clinical patterns is already an area of active investigation in the field of ML/AI in healthcare; programs range from modulating single parameters to advanced predictive modeling to forecast decompensation, among other clinical outcomes, and augment medical decisions (Churpek and Edelson 2016; Kipnis et al. 2016; Brekke et al. 2019). The ongoing COVID-19 health crisis has transformed the aspiration for enhanced clinical decision-making tools into a demand for “Emergency ML.” To support the medical response to COVID-19, researchers are compressing traditional timelines of retrospective training and testing, prospective validation, incremental launches, and deployment of algorithms. Clinical outcomes that an ML algorithm could predict resemble a perpetually moving target. The clinical data, including multiple patient cohorts and a variety of outcomes, are updated daily or more frequently with evolving statistics and ever-increasing observations (Santosh 2020). Since the phenotype of COVID-19 deviates from typical ARDS or other acute organ dysfunctions, existing models of decompensation, risk of mortality, or clinical trajectory prediction created for non-COVID-19 cohorts are not guaranteed to maintain previously reported performance (Wang et al. 2019; Zampieri et al. 2019; Tomasev et al. 2019; Mousavizadeh and Ghasemi 2020). Some currently proposed diagnostic models to detect COVID-19 infection in symptomatic patients excluded proportions of patients with the disease or favored certain predictors, therefore losing key information and potentially limiting performance in widespread screenings (Meng et al. 2020; Song et al. 2020a; Lopez-Rincon et al. 2020; Batista et al. 2020). Thus, adapting and training algorithms on this unique patient population is essential to construct effective prediction models.

**Fig. 1:**
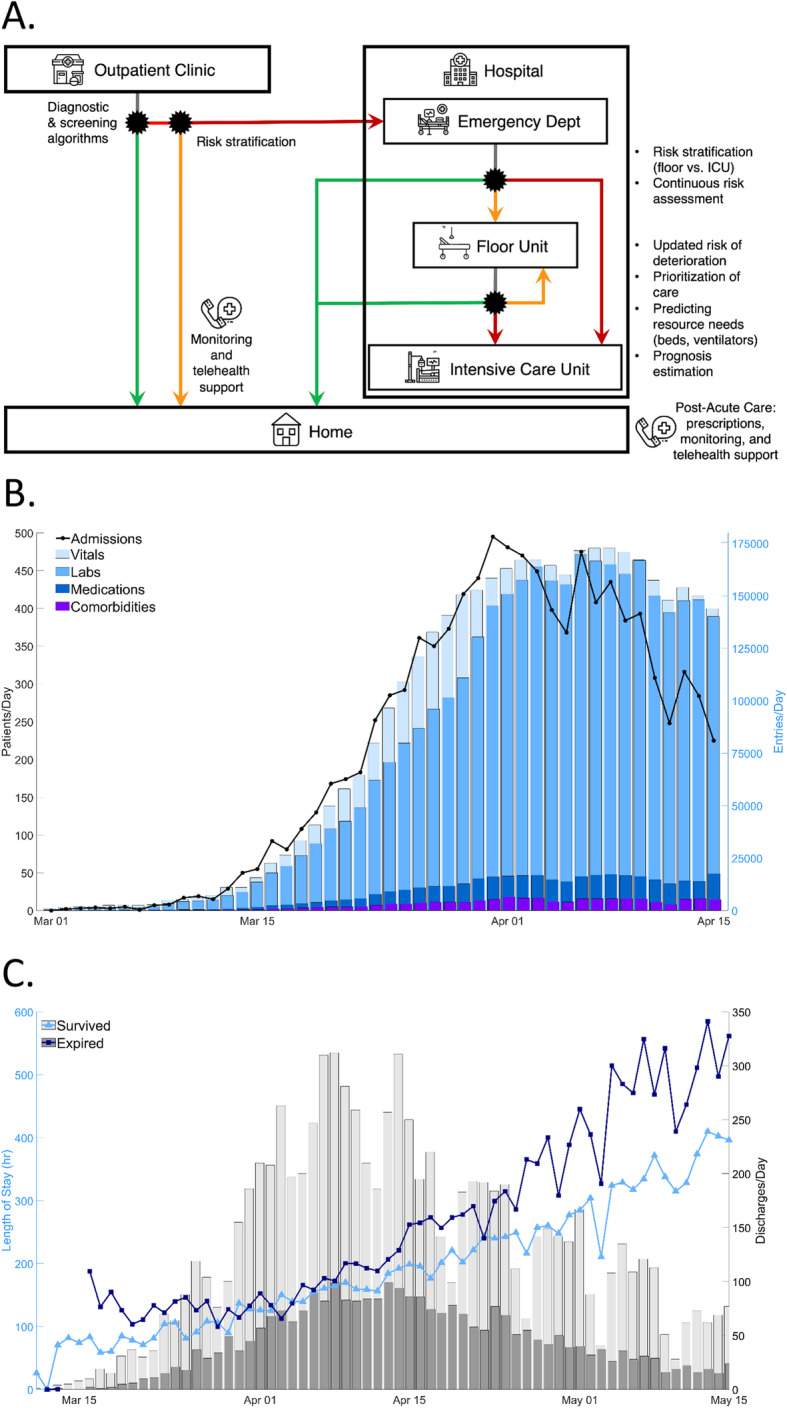
**a** ML/AI in the patient care pathway**.** The black asterisks represent multiple decision points during the patient care pathway that could be augmented by ML/AI tools. The green traces represent a COVID-19 negative diagnosis or recovery while the orange and red traces represent risk stratification of patients by lower and higher risks of deterioration, respectively, as determined by a potential ML/AI model. Additional decisions in the hospital include prioritization of care, allocation of resources, and estimation of prognosis. **b** An expanding COVID-19 database. Since March 1, 2020, there has been an increasing amount of COVID-19 patient data, shown here by new admissions and new medical data entries at Northwell Health, New York’s largest health system, facilities. Given increasing hospital admissions (black trace, left y-axis), there have been hundreds of thousands of new data entries per day (colored bars, right y-axis), including vitals, laboratory results, medication orders, and patient comorbidities. This vast data allows a unique opportunity to implement ML/AI to support medical frontlines and healthcare administrators in the fight against COVID-19. **c** Evolving patient profiles and discharge rates. Basic characteristics of the patient population changed during the progression of the wave of new cases, which can affect performance of a predictive model. For example, the average length of stay for expired patients and those discharged alive (dark blue with square markers and light blue with triangle markers, respectively, left y-axis) diverged in mid-April. Because of these changes, a predictive model with good early performance may decline because of differences between patients hospitalized for three weeks compared to less than a week. Also, an individual’s patient profile may have evolved significantly from hospital admission to those timepoints later during hospitalization. Discharges per day (grey bars, right y-axis) increased with the pandemic’s peak and declined with reduced numbers of new cases

As patients move between a range of clinical settings (outpatient clinics, EDs, floor units, ICUs), they generate unique personal datasets that reflect their phenotypes and require varying clinical resources and participate in multiple decisions, ranging from trivial to potentially life altering. In these settings and during each transition, ML/AI can help clinicians, patients, and their families efficiently process all available data to generate informed, evidence-based recommendations and participate in shared decision-making to identify the optimal course of action. In the age of COVID-19, this can be incorporated into several opportunities across the spectrum of care (Fig. 1a).

The first opportunity lies during initial screening and evaluation of symptomatic people in outpatient facilities. Development of ML models to risk-stratify these individuals and separate low-risk patients from those at higher risk for deterioration can enable focus on the needs of approximately 15% that require more resource-intensive care (Center for Disease Control and Prevention (CDC) COVID-19 Response Team 2020). Identification of low-risk patients can lead to increased utilization of telehealth and virtual care to avoid unnecessary hospital admissions (Dorsey and Topol 2016). Previous ML models have already been developed to reduce avoidable initial admissions (Ngo et al. 2019), predict risk of 30-day readmissions (Frizzell et al. 2017; Golas et al. 2018), and improve pharmaceutical prescriptions (Ribers and Ullrich 2019). Even disease diagnosis can be possible using emerging ML/AI technology; Parkinson’s Disease can be successfully detected by a smartphone-based monitoring platform that extracts features from voice, gait, and reaction time data (Zhan et al. 2018). These approaches suggest the possibility of improving COVID-19-screening phone calls and follow-up survey information (Rao and Vazquez 2020). A model could predict the probability of a confirmed COVID-19 diagnosis and its severity by taking answers from symptomatic individuals and amplifying it with clinical information from electronic health records (EHRs), including comorbidities (DeCaprio et al. 2020), complaints, and demographics (including geography). An ML model could even predict levels of dyspnea over the phone with estimations of emotional affect and cough sounds from speech already possible (Fayek et al. 2017; Porter et al. 2019). The severity prediction could also indicate the level of necessary care: self-monitoring, outpatient doctor visit, or ED visit (Greenhalgh et al. 2020). Prediction models can reduce the number of patients that are admitted to already-overloaded hospitals. However, this type of model may be limited by lack of vital clinical information such as oxygen saturation or severity of dyspnea, which is highly subjective via self-report. Additionally, variability in the exact time that patients develop symptoms after exposure imposes additional challenges (Lauer et al. 2020). Finally, specific patient behavior could influence the quality of data labels used for building ML algorithms. For example, individuals may not call because they have no or mild upper respiratory symptoms; studies estimate that over half of COVID-19 infections are undocumented (Li et al. 2020a). Conversely, individuals may be advised to self-monitor but seek treatment, regardless. These groups could lead to inaccurate labeling for training and testing ML algorithms; more widespread testing and big data could reduce the effects of this hurdle.

The ED represents a second opportunity to apply ML/AI to a data-rich, time-pressured environment where clinicians are called upon to collect, assimilate, and analyze large amounts of data. This begins at the point of triage, where severity of symptoms and the need for urgent intervention are first assessed, to the continued course of patient evaluation through vitals measurements and laboratory results. The changing status of the patient’s health culminates in the decision to either discharge the patient or admit them to the hospital. If escalation of care in the hospital is necessary, determining the most appropriate environment (i.e., floor or critical care unit) is intimately linked to assessing future risk of deterioration. ML/AI models have been developed to be applied at each of these phases within ED care; in triage, vitals representing illness severity can predict resource needs (Levin et al. 2018; Raita et al. 2019) while risk profiles can be determined as new data is obtained (Janke et al. 2016). Lastly, patient condition while in the ED can be used to calculate risk of deterioration or death (Brajer et al. 2020). For patients with COVID-19, ML/AI can provide decision support at each stage by calculating likelihood of admission at triage, refining risk estimates with real data from clinical evaluation, and predicting the patient’s trajectory as well as the effects of prompt ventilator use. A possible input to these models may be chest X-rays and computed tomography (CT) images from diagnosis and disease progression; CT severity scores have been shown to identify patients with severe cases of COVID-19 (Yang et al. 2020), and diagnostic models based on CT images have been proposed to support diagnosis and monitor progression (Jin et al. 2020a; Song et al. 2020b; Xu et al. 2020; Shan et al. 2020; Wang et al. 2020; Ozturk et al. 2020;Jin et al. 2020, 2020a; Li et al. 2020b; Shi et al. 2020a). Within the ED, assessing the possibility of respiratory failure can help institutions and clinicians prioritize and allocate scarce resources as demand outpaces supply.

A third point of intervention lies in the transition from the ED to the inpatient setting and throughout the patient’s stay. The staggering wave of admissions in this pandemic is overwhelming even the most efficient of hospital frontlines. Under normal conditions, hospitals with a large influx of patients tend to struggle to manage them in a timely manner (Morley et al. 2018). Studies have shown that as the time to see admitted patients increases, so does the risk for adverse events (Eriksson et al. 2017). Any time delay in this crisis can lead to a missed opportunity or limited time to save a life. ML/AI techniques can augment the acumen of healthcare providers and aid hospitals in the Herculean task of managing patient volume by calculating measures that can prioritize patients and potentially decrease adverse events. As patient numbers keep increasing, ML/AI tools, based on ever-increasing ongoing vitals, labs, medications, and orders (Fig. 1b), can be applied to calculate risk scores for multiple time points. Short-term predictions (4–8 h) can be used for nurses and physicians to prioritize care. Mid-term predictions (12–24 h) can help units identify patients with the least likelihood of decompensation; this measure of stability can support decisions by clinicians to adapt care on the path to discharge. Lastly, long-term models (more than 24 h) can help administrators allocate precious resources, such as ventilators, beds, and staffing. Clinically predictive tools already exist to predict mortality risks by calculating a score from Multilobular infiltration, hypo-Lymphocytosis, Bacterial coinfection, Smoking history, hyper-Tension, and Age (MuLBSTA) to separate pneumonia patients into relevant categories of care and guide clinical decisions (Guo et al. 2019), and early work with small patient cohorts of COVID-19 has led to models that identify key clinical characteristics that can predict severe cases (Yan et al. 2020; Jiang et al. 2020; Xie et al. 2020; Bai et al. 2020; Caramelo et al. 2020; Lu et al. 2020; Gong et al. 2020; Shi et al. 2020b). One particular model—the Northwell COVID-19 Survival (“NOCOS”) calculator – was built from demographic, laboratory, clinical, and treatment data of over 5200 inpatients to predict survival probability; seven variables from patient EHRs were identified as early predictors of survival, and the easily comprehensible output of the calculator is being used by clinicians to provide critical decision support (Levy et al. 2020). Finally, ML can assist healthcare providers with the most emotionally difficult conversations with patients and their families: goals of care. By providing an objective measure of mortality risk, ML offers clinicians, patients, and their families essential information in this shared decision process.

While researchers in the field of digital medicine and healthcare ML have built predictive models for decades, the urgency to clinically operationalize them is novel. Practices established to tackle COVID-19 can offer a future path for clinical translation of ML/AI tools. The ultimate goal of any model is to maximize patient outcomes for the greatest number of patients within an acceptable ethical framework. However, the inputs to these models can vary based on availability of equipment or staff; heterogeneity in standards of care; responses to anecdotes, personal communications, and preprinted scientific manuscripts; and evolving government policies and professional society guidelines. Such extreme variability in inputs makes setting a fixed operating point, usually based on clinicians’ acceptability of risk, very difficult. For these reasons, models should be operationalized with frequent and automated re-training and re-validation.

Another extremely important consideration is ensuring that all development studies are scientifically and ethically sound. Firstly, available data only represents patients that are admitted and treated. Data-driven models may magnify current disparities in healthcare because there is already less data to represent those with diminished access to care. This issue of equity can limit generalizability of ML/AI models. Secondly, in an outbreak setting, access to any therapy is pressing but still requires strong proof of efficacy (Lane et al. 2016). In this public health emergency, rapid responses are critical, and testing of COVID-19 vaccines has been fast-tracked to phase I trials for early spring of 2020 (Ahmed et al. 2020; Fauci et al. 2020). This provides a compelling precedent to accelerate the deployment of accurate ML models. If an algorithm is proven on retrospective data, it should be implemented into clinical translation for validation and use as quickly as possible. It is crucial to note, however, that an expedited process for model creation and validation can lead to inaccurate results, as seen by many of the early projection models for the progression of COVID-19. To prevent errors and strengthen the accuracy of models over time, active learning is required, with continued emphases on increased frequency for re-training and re-validation as more data becomes available. Moreover, different types of validations can strengthen the stakeholders’ trust in a model’s predictive performance. Prospective validation can address the model’s stability over time, while external validation can address the model’s clinical portability to various hospitals and geographic locations. Whenever possible, both types of validation should be carried out. This concern is not only specific to COVID-19 related models but any application of healthcare ML/AI.

Specifically, during the COVID-19 pandemic, basic characteristics of the patient population can change during the evolving stages of the patient wave, affecting the performance of a predictive model (Fig. 1c). Throughout April, both expired and discharged surviving patients followed similar rates in discharges per day. However, the average length of stay for these two cohorts increased daily, but also diverged significantly by early April, with the patients that expire after that date presenting a five-fold increase in hospitalization duration, compared to a four-fold increase for discharged surviving patients. Such a massive difference in an integral aspect of the dataset, like length of stay, can have a profound effect in prognostic algorithms of arbitrary horizons; while early on in the pandemic, these algorithms would perform very well, they would naturally degrade later on. This decline is due to both the differences in profile of patients that stayed on average 3 weeks in the hospital compared to those that stay less than a week and internal comparisons per patient, when analyzing their medical profile at the beginning of their hospitalization relative to the end. Figure 1c showcases the unique characteristics of a COVID-19 dataset, as we have captured it from our database. As shown, discharge numbers increased and remained high during the pandemic’s peak.

Other potential challenges regard curating high fidelity inputs that can produce reliable outputs for clinician use. It will be necessary to perform automated data scrubbing to all new data entries and establish efficient deployment of updated algorithms. This goal must be achieved by essential integration into standardized pipelines of EHR platforms. The COVID-19 pandemic has mobilized efforts for increasing patient data availability, but these large amounts of new data require continuous quality checks to ensure that desired outputs are achieved. Without high quality data inputs, any model’s output, especially those that rely on evolving databases to increase accuracy, will suffer from garbage in, garbage out (GIGO). In addition, outputs of any model must be easy to evaluate and factor into traditional medical care. An indexed value could represent a patient priority based on probability of outcome. Priority scores can guide allocation of care, ventilators, and beds as well as designate patients to the appropriate medical departments (White and Lo 2020). Finally, amid imposed lockdowns, maintaining communication between the ML engineers, data scientists, and stakeholders, including hospital frontlines and healthcare administrators, can be challenging. Sustaining increased productivity and morale is necessary for the success of all such endeavors.

## Conclusions

While caring for thousands of COVID-19 patients, hospital staff, nurses, physicians, administrators, scientists, and engineers have also been pursuing ways to optimize care to face the onslaught of daily new cases. ML and AI is becoming more prevalent in healthcare and medicine, and the worldwide COVID-19 crisis presents a critical situation that demands implementation of ML approaches, whether applications are for medical treatment research, patient care, allocating resources, or managing hospital volume. Medical personnel in all clinical settings, including doctors and nurses, need a support system in a shared decision-making process that includes patients and their families. Expanding datasets provide an example of available information in this unique opportunity for deployment of an unprecedented “emergency ML” effort. Northwell Health, New York’s largest health system, serves multiple communities at the world’s current epicenter of the COVID-19 pandemic and maintains an example of a sizeable COVID-19 patient database—featuring vitals, laboratory results, ordered medications, and continuously captured and updated demographic information, and it has already been used to present characteristics and early clinical outcomes of 5700 hospitalized COVID-19 patients in the New York City area (Richardson et al. 2020). With increasing health data, evidence-based prediction tools trained and validated properly and often can guide overwhelmed hospital frontlines and administrators to make informed decisions in a challenging time. With the best possible data and analytics, the field of ML/AI can be a key ally in the fight to limit the devastating consequences of COVID-19.

## Data Availability

The data that support the findings of this study are available on request from COVID19@northwell.edu. The data are not publicly available due to restrictions as it could compromise the privacy of research participants.

## Abbreviations

AI: Artificial intelligence
ARDS: Acute respiratory distress syndrome
COVID-19: Coronavirus disease 2019
CT: Computed tomography
ED: Emergency department
EHR: Electronic health record
ICU: Intensive care unit
ML: Machine learning
MuLBSTA: Multilobular infiltration, hypo-Lymphocytosis, Bacterial coinfection, Smoking history hyper-Tension, and Age
NOCOS: Northwell COVID-19 Survival
SARS-CoV2: Severe acute respiratory syndrome coronavirus 2
